# The First Complete Chloroplast Genome of Spider Flower (*Cleome houtteana)* Providing a Genetic Resource for Understanding Cleomaceae Evolution

**DOI:** 10.3390/ijms26083527

**Published:** 2025-04-09

**Authors:** Lubna, Rahmatullah Jan, Syed Salman Hashmi, Saleem Asif, Saqib Bilal, Muhammad Waqas, Ashraf M. M. Abdelbacki, Kyung-Min Kim, Ahmed Al-Harrasi, Sajjad Asaf

**Affiliations:** 1 Natural and Medical Science Research Center, University of Nizwa, Nizwa 616, Omansshashmi10@gmail.com (S.S.H.);; 2 Coastal Agriculture Research Institute, Kyungpook National University, Daegu 41566, Republic of Korea; rehmatbot@yahoo.com; 3 Department of Applied Biosciences, Kyungpook National University, Daegu 41566, Republic of Korea; 4 Department of Agriculture Extension, Government of Khyber Pakhtunkhwa, Mardan 23200, Pakistan; agronomist89@gmail.com; 5 Deanship of Skills Development, King Saud University, P.O. Box 2455, Riyadh 11451, Saudi Arabia

**Keywords:** chloroplast genome, SSRs, *Ycf*1 gene, phylogenetic analysis, repetitive elements, inverted repeat boundaries, nucleotide diversity, selective pressure

## Abstract

In the present study, the sequencing and analysis of the complete chloroplast genome of *Cleome houtteana* and its comparison with related species in the Cleomaceae family were carried out. The genome spans 157,714 base pairs (bp) and follows the typical chloroplast structure, consisting of a large single-copy (LSC) region (87,506 bp), a small single-copy (SSC) region (18,598 bp), and two inverted repeats (IRs) (25,805 bp each). We identified a total of 129 genes, including 84 protein-coding genes, 8 ribosomal RNA (rRNA) genes, and 37 transfer RNA (tRNA) genes. Our analysis of simple sequence repeats (SSRs) and repetitive elements revealed 91 SSRs, with a high number of A/T-rich mononucleotide repeats, which are common in chloroplast genomes. We also observed forward, palindromic, and tandem repeats, which are known to play roles in genome stability and evolution. When comparing *C. houtteana* with its relatives, we identified several highly variable regions, including *ycf*1, *ycf*2, and *trn*H–*psb*A, marking them as propitious molecular markers for the identification of species as well as phylogenetic studies. We examined the inverted repeat (IR) boundaries and found minor shifts in comparison to the other species, particularly in the *ycf1* gene region, which is a known hotspot for evolutionary changes. Additionally, our analysis of selective pressures (Ka/Ks ratios) showed that most genes are under strong purifying selection, preserving their essential functions. A sliding window analysis of nucleotide diversity (Pi) identified several regions with high variability, such as *trn*H–*psb*A, *ycf*1, *ndh*I*-ndh*G, and *trn*L-*ndh*F, highlighting their potential for use in evolutionary and population studies. Finally, our phylogenetic analysis, using complete chloroplast genomes from species within Cleomaceae, Brassicaceae, and Capparaceae, confirmed that *C. houtteana* belongs within the Cleomaceae family. It showed a close evolutionary relationship with *Tarenaya hassleriana* and *Sieruela rutidosperma*, supporting previous taxonomic classifications. The findings from the current research offer invaluable insights regarding genomic structure, evolutionary adaptations, and phylogenetic relationships of *C. houtteana*, providing a foundation for future research on species evolution, taxonomy, and conservation within the Cleomaceae family.

## 1. Introduction

The Cleomaceae family, which is split into approximately 18 genera and 150 to 200 species, exists in a variety of tropical and subtropical zones across the globe [1]. Like many other families, it was formally included in the Capparaceae family due to certain phenotypic resemblances, especially concerning floral and fruiting processes. However, Cleomaceae has been proven by molecular phylogenetics studies utilizing nuclear and plastid DNA sequences to represent a distinct unit of evolution stronger than that of Brassicaceae [2]. This rearrangement under Capparidinieae greatly enhances the understanding of the evolutionary complexities within the Cleomaceae family and, correlatively, the entire order, which includes species with major economic significance such as *Brassica oleracea*, *Arabidopsis thaliana*, and *Raphanus sativus* [3].

The genus Cleome is considered the most varied in terms of species within the Cleomaceae family, with about 180–200 species undergoing description, many of which have different forms of growth, including annual and perennial herbs and shrubs [4]. Some species in this genus were analyzed for their adaptability to the environment and for their ethno-medicinal applications. A number of Cleome species exhibit antimicrobial, antioxidant, and insecticidal activities because they possess secondary metabolites, which include glucosinolates, flavonoids, alkaloids, and terpenoids. Though these species have important ecological and pharmacological aspects, the taxonomic connections among them are still unresolved due to a lack of identification and insufficient molecular data evidence. *Cleome houtteana* is notable among these species, which has been grown extensively for its ornamental purposes and is commonly confused with other morphologically similar taxa like *Cleome spinosa* and *Tarenaya hassleriana* [1,5]. Such cases of confusion underline the necessity for molecular markers that can accurately delineate species within the Cleomaceae family.

In Pakistan, where *C. houtteana* is found in semi-arid and arid ecological zones, it holds great environmental value. This species is so remarkably beautiful that it can be seen growing both in untouched natural beauty and in cultivated gardens and fields [6]. Apart from its ornamental use, *C. houtteana* has significant ecological importance as it attracts bees and butterflies for pollination, which helps in saving wildlife. It is also important in medicine because traditional herbal practitioners in Pakistan have used it for a variety of diseases, such as inflammation, skin disorders, and problems with the digestive system. The plant has bioactive substances like alkaloids and flavonoids, which, according to the literature, have a positive effect as an antioxidant and antimicrobial [7].

The plant systematics domain has been revolutionized by the development of tools for sequencing the chloroplast genome since it outlines an authentic molecular basis to solve phylogenetic problems. The chloroplast genomes possess a highly conserved quadripartite structure that ranges between 120 and 170 kb in size, comprising a large single-copy (LSC) region, a small single-copy (SSC) region, and two inverted repeat (IR) regions [8,9,10]. Biogeographic and evolutionary studies, species identification, and even genomic resource analyses are perfectly possible with the maternal inheritance of most angiosperms and the lack of recombination within the chloroplast genome [11]. In the last decade, complete chloroplast genome sequencing has been extensively utilized to reconstruct evolutionary histories, analyze genetic diversity, and create molecular markers for taxonomic identification [12,13,14].

Research has shown through comparative genomic analyses that species from the family Cleomaceae have a varying range of structural differences in their chloroplast genomes like the loss of genes, the inversion of repeat boundaries, and the expansion or contraction of intergenic spacer regions [15]. Previous research on *Cleome chrysantha* and *Dipterygium glaucum* generated results for what could be termed as “divergence hotspots” useful in phylogenetic research [15]. Also, phylogenetic reconstructions provided with complete sequences of the chloroplast genome demonstrated that Cleomaceae is monophyletic, separate from Capparaceae, which provides more evidence for its familial independence [1,4]. The chloroplast genome of *C. houtteana* remains unsaid and unresearched, thus positioning ourselves critically in the understanding of the evolutionary development and genomic structure of the species. Other angiosperm families have shown the successful resolution of these taxonomic issues from the whole-chloroplast genome sequencing approach; hence, applying it to *C. houtteana* would most likely resolve the phylogenetic puzzles and provide clues about genome evolution in Cleomaceae.

This study compares the complete chloroplast genome sequence of *C. houtteana* with the other members of the Cleomaceae family and its relatives. Our goals are to identify the structural differences and divergences within the Cleomaceae family and their corresponding phylogenetic relationships. We attempt to differentiate *C. houtteana* from its congeners by analyzing codon usage bias, simple sequence repeats (SSRs), and modifying hotspots of sequence variation. Additionally, we determine its place within Cleomaceae and its relationship with Brassicaceae by performing phylogenetic reconstructions with complete chloroplast genome datasets. These comparisons may lead to the refinement of the taxonomy of Cleomaceae, the development of new molecular identification markers, and the enhancement of genomic resources for studies on the evolution and ecology of the family.

## 2. Results

### 2.1. Chloroplast Genome Sequencing and Comparison

**Figure 1:**
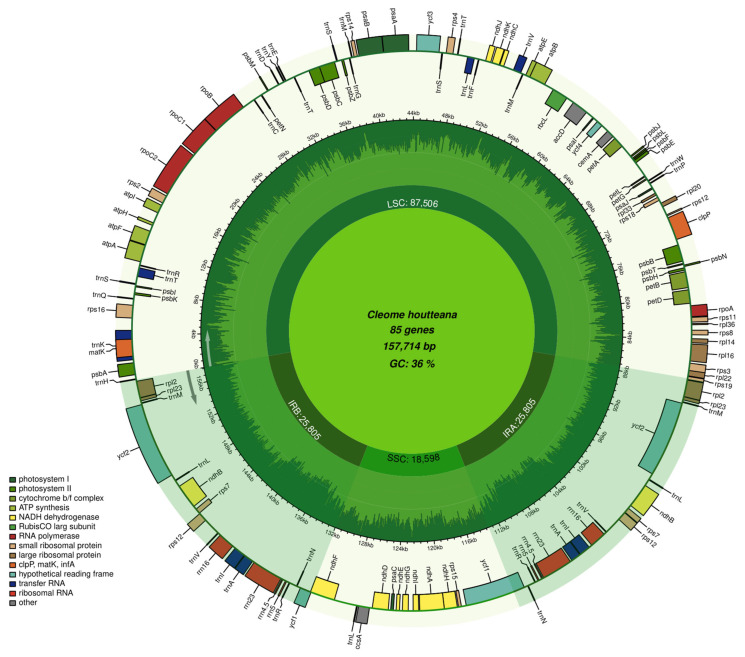
Genome map of the *C. houtteana* plastome. The IR regions are shown in dark colors, dividing the chloroplast genome into large (LSC) and small (SSC) single-copy regions. Genes inside the circle are transcribed clockwise, while those outside are transcribed counterclockwise. Genes belonging to different functional groups are color-coded. The inner ring represents GC content (light green) and AT content (dark green), with a legend added for clarity. A genome length scale (in kb) is included around the outer circle to provide a size reference. The circular chloroplast genome map was generated using Chloroplot (https://irscope.shinyapps.io/Chloroplot/, accessed on 10 November 2024).

The complete chloroplast genome of *C. houtteana* was successfully sequenced for the first time and compared with closely related species from the Cleomaceae family. The total genome length of *C. houtteana* was determined to be 157,714 bp, which falls within the size range observed among related species, varying from 154,124 bp in *Cl. lutea* to 159,393 bp in *Coalisina paradoxa* (Table 1). The GC content of *C. houtteana* was found to be 35.8%, consistent with most Cleomaceae members except for *Cleome chrysantha* and *Cleomella lutea*, which exhibited slightly higher GC contents of 36.0% and 36.5%, respectively (Table 1). The genome is structured into an LSC region of 87,506 bp, an SSC region of 18,598 bp, and two IRs, each measuring 25,805 bp. Minor variations in LSC, SSC, and IR sizes were observed across species, likely due to expansion/contraction events in the IR boundaries, a common evolutionary process in angiosperm chloroplast genomes (Figure 1).

### 2.2. Gene Annotation and Comparison

In total, 129 genes were annotated in *C. houtteana*, which included 84 protein-coding genes, 37 tRNA genes, and 8 rRNA genes, with 16 genes containing introns. The number of genes was comparable to most species in the dataset, with values ranging between 129 and 134. Notably, *C. pallida* had the largest number of total protein-coding genes (134 and 87, respectively), while *C. houtteana* was similar to *G. gynandra* with 129 total genes. The protein-coding DNA (PCD) size was 76,590 bp, which was slightly lower than *C. chrysantha* (79,488 bp) and *C. pallida* (80,076 bp), indicating minor differences in gene lengths and intergenic regions. The number of genes with introns was highest in *C. houtteana* (16 intron-containing genes) along with *Co. paradoxa* (16), whereas *C. chrysantha* exhibited the lowest count (11), suggesting potential intron loss events in certain species (Table 1 and Figure 1).

The functional annotation of *C. houtteana* revealed that the chloroplast genome encodes genes involved in photosynthesis, self-replication, and other essential metabolic functions, along with a set of conserved open reading frames (*ycf* genes) with unknown functions (Table 2). Photosynthesis-related genes include ATP synthase subunits (*atp*A, *atp*B, *atp*E, *atp*F, *atp*H, and *atp*I), photosystem I (*psa*A, *psa*B, *psa*C, *psa*I, and *psa*J) and II subunits (*psb*A–*psb*Z, and *ycf*3), the cytochrome b6/f complex (*pet*A, *pet*B, *pet*D, *pet*G, *pet*L, and *pet*N), NADH dehydrogenase (*ndh*A–*ndh*K), and the Rubisco large subunit (*rbc*L), all of which play critical roles in light energy capture and electron transport.

Self-replication genes ensure the independent maintenance and function of the chloroplast genome. These include ribosomal proteins (*rpl*14, *rpl*16, *rpl*2, *rpl*20, *rpl*22, *rpl*23, *rpl*33, and *rpl*36 and *rps*11, *rps*12, *rps*14, *rps*15, *rps*16, *rps*18, *rps*19, *rps*2, *rps*3, *rps*4, *rps*7, and *rps*8), as well as RNA polymerase subunits (*rpo*A, *rpo*B, *rpo*C1, and *rpo*C2), which transcribe plastid genes. Additional genes encode functions beyond photosynthesis and self-replication, including *acc*D for lipid metabolism, *ccs*A for cytochrome c synthesis, *cem*A for membrane transport, *clp*P for protein degradation, and *mat*K, a maturase enzyme involved in intron splicing. The presence of four highly conserved ORFs (*ycf*1, *ycf*2, *ycf*3, and *ycf*4) suggests additional functional roles yet to be fully elucidated (Table 2). While *ycf*3 has been implicated in photosystem I assembly, the functions of *ycf*1, *ycf*2, and *ycf*4 remain ambiguous but are considered essential for chloroplast development and genome stability.

### 2.3. Intron–Exon Structure of C. houtteana Chloroplast Genes

The intron–exon organization of the *C. houtteana* chloroplast genome was meticulously analyzed, revealing significant variations in exon and intron lengths across different genes. A total of 18 genes were identified as containing introns, a finding that aligns with prior observations of chloroplast genomes within the Cleomaceae family (Table 3). These intron-containing genes encompass a diverse functional spectrum, including tRNAs, ribosomal proteins, ATP synthase subunits, polymerases, and NADH dehydrogenase subunits.

Among the intron-bearing genes, the longest exon was observed in the *rpo*C1 gene, measuring 1611 bp, while the shortest exon was identified in *rpl*16, with a length of merely 9 bp. This substantial variation in exon sizes underscores the complexity of the chloroplast genome. The largest intron was detected in the *trn*K-UUU gene, spanning 2564 bp, followed by *ndh*A with an intron of 1126 bp (Table 3). The presence of large introns suggests potential regulatory roles, such as alternative splicing or post-transcriptional modifications, which may be crucial for gene expression regulation.

Several genes exhibited a two-intron structure, notably *ycf*3 and *clp*P. *ycf*3, which is involved in photosystem I assembly, contains three exons separated by two introns of varying lengths: exons of 124 bp, 230 bp, and 153 bp, with introns measuring 707 bp and 804 bp. Similarly, *clp*P, a protease gene essential for protein degradation, has exons of 71 bp, 294 bp, and 235 bp, with introns measuring 901 bp and 596 bp. This complex transcript processing highlights the intricate regulatory mechanisms at play in chloroplast gene expression.

The presence of introns in self-replicative genes, such as *rpl*2, *rpl*16, and *rps*16, underscores the evolutionary conservation of splicing mechanisms essential for ribosomal function. Transfer RNA genes, including *trn*K-UUU, *trn*L-UAA, *trn*V-UAC, *trn*A-UGC, and *trn*E-UUC, also contained introns, reinforcing the importance of splicing in chloroplast tRNA maturation. These findings indicate that intron-containing genes are highly conserved in *C. houtteana*, with structural variations contributing to chloroplast genome stability and expression regulation.

### 2.4. Codon Usage Analysis of C. houtteana Chloroplast Genome

The codon usage analysis of the *C. houtteana* chloroplast genome revealed a pronounced preference for A/T-ending codons, which is consistent with the AT-rich composition characteristic of chloroplast genomes. Among the 61 codons encoding the 20 standard amino acids, Leucine (L), Isoleucine (I), and Serine (S) were identified as the most frequently encoded amino acids. The most abundant codons were TTT (Phenylalanine, 40.34%, 1496 occurrences), ATT (Isoleucine, 45.27%, 1679 occurrences), and GAA (Glutamic Acid, 38.75%, 1437 occurrences), all of which play pivotal roles in the structure and function of chloroplast-encoded proteins (Table S1).

A strong bias was observed in synonymous codon usage, favoring A/T-ending codons over G/C-ending ones. For instance, GAT (Aspartic Acid) was used 28.01% of the time (1039 occurrences), compared to GAC (6.95%, 258 occurrences), indicating a preference for transcriptionally efficient codons. Stop codon usage also exhibited a bias, with TAA (4.23%) being the most frequently used, followed by TAG (3.18%) and TGA (2.91%), ensuring efficient translation termination (Table S1).

### 2.5. Repeat Sequences in C. houtteana and Related Cleomaceae Species

**Figure 2:**
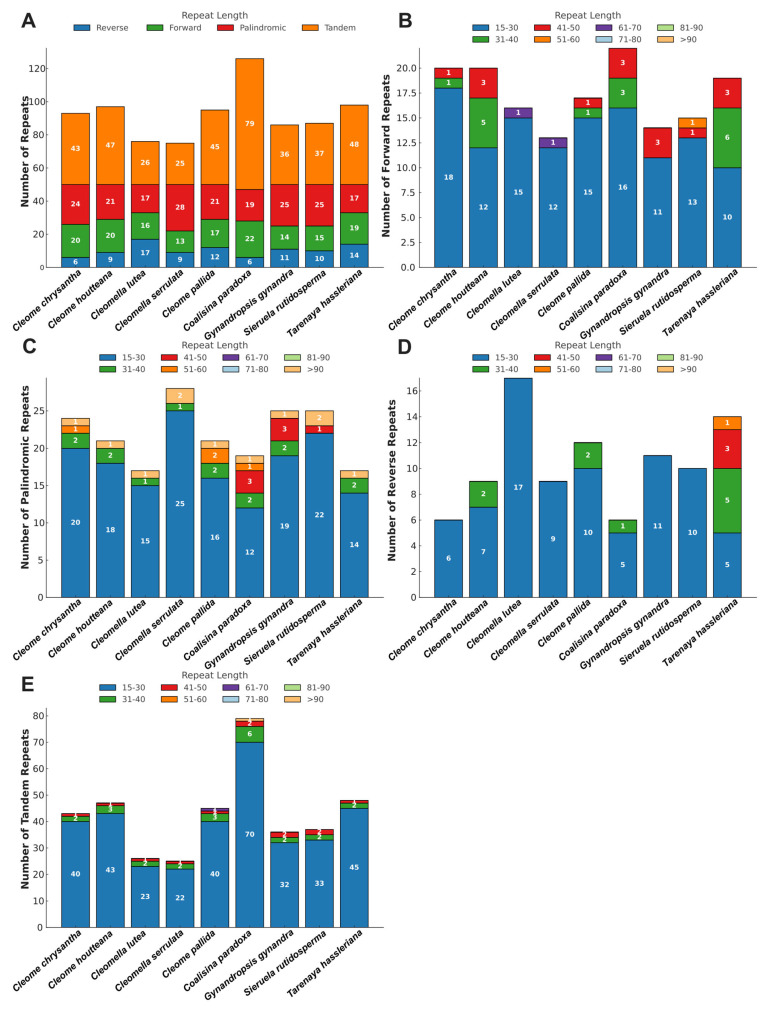
Repetitive sequences in *C. houtteana* and eight related plastomes: (**A**) total number of repetitive sequences. (**B**) lengthwise frequency of forward repeats in plastomes; (**C**) lengthwise frequency of palindromic repeats; (**D**) lengthwise frequency of reverse repeats; (**E**) lengthwise frequency of tandem repeats.

The analysis of repeat sequences in the chloroplast genome of *C. houtteana* and its relatives within the Cleomaceae family unveiled variations in the distribution and frequency of forward, palindromic, reverse, and tandem repeats. These repeats are instrumental in maintaining genome stability, facilitating recombination, and driving structural variation among plastid genomes. The total number of forward repeats in *C. houtteana* was 20, which is similar to *C. chrysantha* (20) but slightly higher than *C. pallida* (17) and *Cl. serrulata* (13) (Figure 2). Most forward repeats were within the 15–30 bp range, with *C. houtteana* exhibiting 12 such repeats, which is fewer than *C. chrysantha* (18) but more than *Cl. serrulata* (12). The presence of only a few longer repeats (31–50 bp) suggests that forward repeat-mediated genome expansion is not a dominant evolutionary mechanism in Cleomaceae (Figure 2).

Palindromic repeats were more abundant than forward repeats across all analyzed species, indicating their importance in stabilizing the chloroplast genome by preventing recombination errors. *C. houtteana* contained 21 palindromic repeats, a number slightly lower than *Cl. serrulata* (28) but comparable to *C. chrysantha* (24) and *C. pallida* (21) (Figure 2C). The majority of these repeats were in the 15–30 bp range, with *C. houtteana* containing 18, which is slightly less than *Cl. serrulata* (25) but more than *C. pallida* (16). Longer palindromic repeats (>90 bp) were rare, with only a single occurrence in *C. houtteana* and most other species. This distribution suggests a selective constraint maintaining palindromic repeats within a limited size range, which is likely to prevent excessive recombination activity that could destabilize the chloroplast genome.

Reverse repeats were the least frequent among the repeat types analyzed, with *C. houtteana* containing 9, a count higher than *C. chrysantha* (6) and *Cl. serrulata* (9) but significantly lower than *Cl. lutea* (17). The majority of reverse repeats in *C. houtteana* fell within the 15–30 bp range, which is consistent with the pattern observed in other species (Figure 2D). Only a few were within the 31–40 bp range, similar to *C. pallida*, indicating that large-scale structural variations facilitated by reverse repeats are uncommon in Cleomaceae. The lower abundance of reverse repeats, compared to palindromic and forward repeats, aligns with previous findings in plastid genomes, where reverse repeats are less favored due to their potential to induce rearrangements that compromise genome integrity.

Tandem repeats were the most abundant among all repeat categories, suggesting their significant role in shaping plastid genome structure. *C. houtteana* exhibited 47 tandem repeats, within the observed range for Cleomaceae species. Most of these repeats were within the 15–30 bp range, with *C. houtteana* having 43, closely matching *C. chrysantha* (40) and consistent with the preference for short tandem repeats in plastid genomes (Figure 2E). Longer tandem repeats (>40 bp) were rare across all species, indicating selective constraints against the expansion of these repeat elements. Short tandem repeats are known to contribute to genome plasticity, transcriptional regulation, and evolutionary divergence, reinforcing their significance in chloroplast genome evolution.

### 2.6. SSRs in C. houtteana and Related Cleomaceae Species

**Figure 3:**
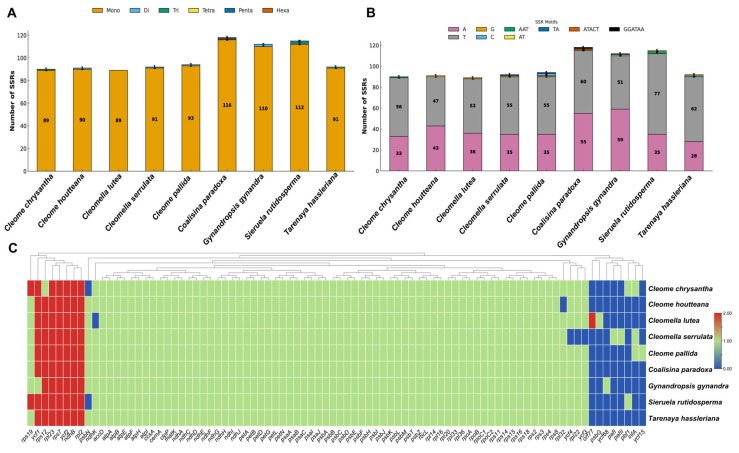
Analysis of the simple sequence repeats (SSRs) in *C. houtteana* and eight related plastomes: (**A**) total number of SSRs in genomes; (**B**) frequency of the simple sequence repeat motif in the chloroplast genome of *C. houtteana* and eleven related plastomes; (**C**) summary of genes lost across *C. houtteana* and related species plastomes. The blue color shows the missing genes, the green color shows single genes, and the red color shows the genes duplicated in plastomes.

The analysis of SSRs in the chloroplast genome of *C. houtteana* and related Cleomaceae species revealed variations in SSR abundance and motif composition. SSRs, also known as microsatellites, are short tandem repeats that play a significant role in genome evolution, recombination, and genetic diversity. The total number of SSRs in *C. houtteana* was 91, which is comparable to *C. chrysantha* (90) and *Cl. lutea* (89) but slightly lower than *C. pallida* (94) and *Cl. serrulata* (92), indicating minor variations in SSR expansion across species (Figure 3A).

Mononucleotide repeats were the most abundant SSR type in all analyzed species, with *C. houtteana* containing 90 such repeats, which is similar to *C. chrysantha* (89) and *Cl. lutea* (89) but less than *C. pallida* (93). Dinucleotide repeats were rare, with *C. houtteana* and *Cl. serrulata* containing only one each, whereas *C. chrysantha* and *Cl. lutea* lacked dinucleotide repeats altogether (Figure 3A). Trinucleotide repeats were nearly absent, with only a single occurrence in *C. chrysantha* and none in *C. houtteana* or other species. No tetranucleotide, pentanucleotide, or hexanucleotide repeats were detected in *C. houtteana*, and these were absent in most other species as well, suggesting a strong evolutionary constraint against the expansion of complex SSR motifs in chloroplast genomes.

The predominance of mononucleotide SSRs, particularly A/T-rich motifs, is consistent with the AT-biased composition of chloroplast genomes and suggests a preference for mutational events that contribute to genome plasticity. The presence of a single dinucleotide repeat in *C. houtteana* and other species highlights the rarity of these motifs, potentially due to selective pressures limiting their expansion in plastid genomes (Figure 3B). The overall SSR profile of *C. houtteana* closely resembles that of other Cleomaceae species, indicating a strong evolutionary conservation of SSR distribution patterns in chloroplast genomes. Differences in total SSR counts and motif compositions among species may reflect subtle genomic adaptations or lineage-specific variations in replication slippage events.

### 2.7. Gene Presence, Absence, and Duplication in C. houtteana and Related Species

The gene content of the *C. houtteana* chloroplast genome was compared with some of the closely related Cleomaceae species to assess gene presence, absence, and duplication events. The analysis revealed a high degree of conservation in core chloroplast genes, with most species exhibiting a nearly identical set of essential genes involved in photosynthesis, self-replication, and metabolic processes. The majority of genes, including *acc*D, *atp*A, *atp*B, *atp*E, *atp*F, *atp*H, *atp*I, *ccs*A, and *cem*A, were present in all species analyzed, indicating strong evolutionary constraints maintaining chloroplast genome functionality (Figure 3C).

Gene duplication events were observed in several species, particularly in *rps*7, *ycf*1, and *ycf*2. *C. houtteana*, along with *C. chrysantha*, *Cl. lutea*, and *C. pallida*, exhibited duplication of *rps*7 and *ycf*2, whereas *Cl. serrulata* lacked the duplicated *ycf*3, highlighting a potential divergence in genome structure. The presence of two copies of *ycf*1 in *C. houtteana* and other species suggests a conserved duplication pattern within Cleomaceae, as *ycf*1 is known to play a role in chloroplast genome stability. However, *ycf*15 was absent in all species except *C. pallida*, suggesting a possible pseudogenization or lineage-specific loss event (Figure 3C).

The presence of certain genes varied across species, with *ycf*4 missing in *Cl. serrulata* but retained in all other species. Similarly, *ycf*68 was absent in all species analyzed, indicating that it may not be essential for chloroplast function in Cleomaceae. The overall gene presence and duplication patterns suggest that while the chloroplast genome of *C. houtteana* remains highly conserved, minor variations in gene duplications and losses reflect evolutionary adaptations unique to specific lineages.

### 2.8. Gene Divergence in C. houtteana and Related Chloroplast Genomes

**Figure 4:**
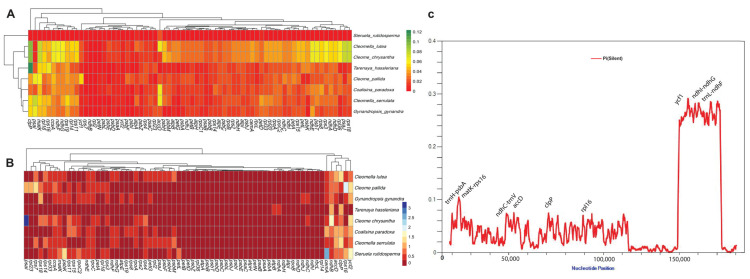
(**A**) Heatmap showing pairwise sequence distance of 70 genes from *C. houtteana* and related plastomes. (**B**) pairwise ratios of non-synonymous rates (Ka) to synonymous rates (Ks) in *C. houtteana*. This heatmap illustrates the Ks/Ks ratios for 77 protein-coding genes across nine species from the Cleomaceae family. (**C**) Sliding window analysis of nucleotide variability among *C. houtteana* and related plastomes (window length: 600 bp; step size: 100 bp).

The divergence analysis of shared genes in Cleomaceae chloroplast genomes, with *C. houtteana* as the reference, revealed varying levels of sequence divergence among species. Overall, the data demonstrated that essential photosynthetic and self-replication genes exhibited strong conservation, while certain genes showed moderate divergence, indicating potential evolutionary adaptation. Among the compared genomes, *Cl. lutea* and *Cl. serrulata* exhibited the highest sequence divergence from *C. houtteana*, with genes such as *acc*D showing divergence values of 0.0653 and 0.0653, respectively, as depicted in Figure 4A, suggesting that the *acc*D gene, which is involved in fatty acid biosynthesis, has undergone significant evolutionary changes in these species. Similarly, genes such as *cem*A (encoding a membrane-associated protein) and *clp*P (a protease gene) exhibited higher divergence values in *Cl. lutea* (0.0403 and 0.0933, respectively) compared to *Cl. serrulata* (0.0211 and 0.0538), indicating varying selective pressures in different species (Figure 4A).

*Co. paradoxa* showed moderate divergence from *C. houtteana*, with *acc*D (0.0652) and *cem*A (0.0226) exhibiting notable differences. However, core genes such as *rps*16 (0.0265) and *rps*3 (0.0236) remained relatively conserved, suggesting that ribosomal protein-coding genes are under strong purifying selection. In contrast, *S. rutidosperma* exhibited the lowest levels of divergence, with several genes (*atp*E, *atp*H, *atp*I, *cem*A, *clp*P, *rps*16, *rps*18, *rps*19, *rps*2, *ycf*1, and *ycf*2) showing negligible divergence values (0.0000 to 0.0025), indicating an extremely close evolutionary relationship with *C. houtteana*.

*G. gynandra*, while closely related to *C. houtteana*, showed moderate divergence in genes such as *acc*D (0.0212), *cem*A (0.0149), and *clp*P (0.0542), highlighting species-specific evolutionary patterns. The *ycf*1 and *ycf*2 genes, which are often involved in plastid genome stability, exhibited relatively low divergence across all species, reinforcing their functional importance.

### 2.9. Selective Pressure (Ka/Ks) and Nucleotide Diversity (Pi) in C. houtteana and Related Chloroplast Genomes

The non-synonymous to synonymous substitution ratio (Ka/Ks) was analyzed for shared genes in Cleomaceae chloroplast genomes using *C. houtteana* as the reference. The Ka/Ks ratio provides insights into selective pressures acting on genes, where values <1 indicate purifying selection, values =1 suggest neutral evolution, and values >1 imply positive selection. To statistically validate selection signals, we performed Fisher’s Exact Test for genes with Ka/Ks > 1 and reported *p*-values to assess significance.

The highest Ka/Ks values were observed in *acc*D, *cem*A, and *clp*P, suggesting relaxed purifying selection or potential adaptive evolution in these genes. *Cl. lutea* exhibited a Ka/Ks of 0.926 for the *acc*D gene, indicating that this gene has undergone significant evolutionary change in this species. *C. pallida* showed a Ka/Ks of 0.655 for *acc*D, while *G. gynandra* exhibited a lower value of 0.306, suggesting stronger purifying selection in the latter (Figure 4B). Similarly, *cem*A, which encodes a chloroplast envelope membrane protein, displayed relatively high Ka/Ks values across species, reaching 0.490 in *Cl. lutea*, 0.483 in *C. pallida*, and 0.398 in *G. gynandra*. This pattern suggests that *cem*A may be subject to functional diversification in different lineages.

The protease gene *clp*P, essential for protein degradation, exhibited the highest Ka/Ks ratios among all genes analyzed, with *Cl. lutea* showing a Ka/Ks of 1.210. However, Fisher’s Exact Test did not yield a statistically significant *p*-value, suggesting that while the gene may experience relaxed selection, there is insufficient evidence to confirm strong positive selection. Similarly, *C. pallida* and *C. chrysantha* had Ka/Ks values of 0.532 and 0.651, respectively, supporting relaxed selection rather than definitive adaptive evolution (Figure 4B).

Ribosomal protein genes (*rps*7, *rps*8, and *rps*16) generally exhibited moderate Ka/Ks values, suggesting functional conservation under purifying selection. However, *rps*7 in *C. pallida* showed a Ka/Ks of 1.309. While this could indicate adaptive changes, Fisher’s Exact Test did not provide statistical significance, suggesting that further analysis is required to confirm positive selection. The *ycf*2 gene, often involved in plastid genome stability, had high Ka/Ks values in C. pallida (1.279) and C. chrysantha (1.473), but statistical tests failed to confirm significant positive selection (*p* > 0.05). This suggests that *ycf*2 may be experiencing functional divergence rather than strong adaptive evolution.

Overall, our analysis reveals that most chloroplast genes are subject to strong purifying selection, maintaining their essential functions. However, genes such as *acc*D, *cem*A, *clp*P, and *ycf*2 exhibit higher Ka/Ks values in certain species, suggesting lineage-specific evolutionary pressures. While some genes (e.g., *rps7*, *clp*P, and *ycf*2) show elevated Ka/Ks values, the absence of statistically significant *p*-values indicates that positive selection remains inconclusive and requires further validation.

The nucleotide diversity (Pi) of the *C. houtteana* chloroplast genome was analyzed alongside eight related Cleomaceae species using DnaSP software (version 6.13.03) with a sliding window of 600 bp and a step size of 100 bp. The results revealed distinct patterns of nucleotide variation, reflecting evolutionary pressures on coding and non-coding regions. Non-coding regions, particularly intergenic spacers such as *trn*H–*psb*A, *mat*K-*rps*16, ndhI-ndhG, and trnL-ndhF, along with hypervariable loci *ycf*1, displayed high nucleotide diversity, indicating hotspots for recombination and evolutionary divergence. Conversely, coding regions, including photosynthetic genes such as *rbc*L, *psa*A, and *psb*B–*psb*D, exhibited low nucleotide diversity, which is consistent with strong purifying selection to maintain essential functions.

### 2.10. IR Contraction and Expansion in C. houtteana and Related Chloroplast Genomes

**Figure 5:**
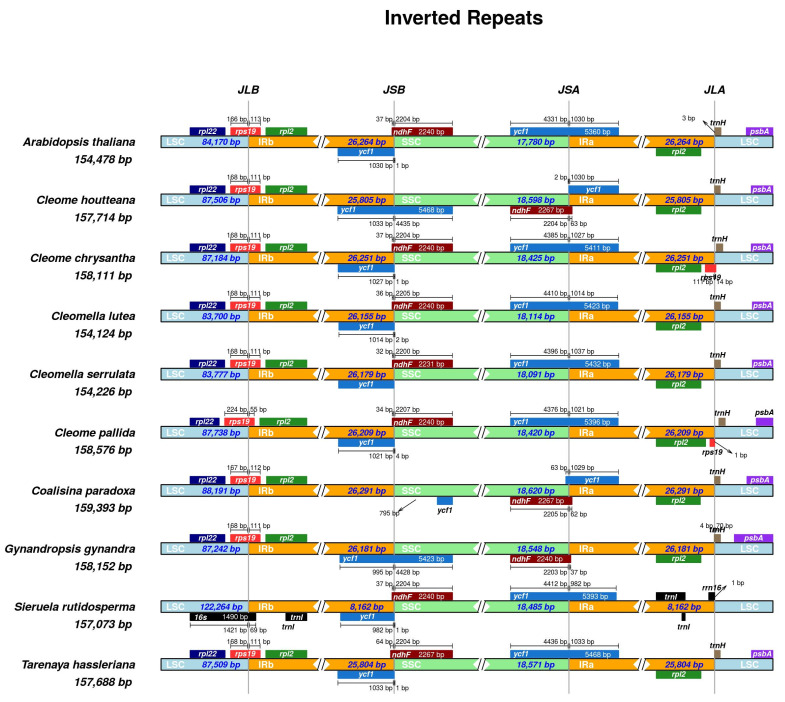
Distances between adjacent genes and junctions of the small single-copy (SSC), large single-copy (LSC), and two inverted repeat (IR) regions among *C. houtteana* and related plastomes. Boxes above and below the primary line indicate the adjacent border genes. The figure is not scaled in terms of sequence length and only shows relative changes at or near the IR/SC borders.

The comparative analysis of IR contraction and expansion in *C. houtteana* and related Cleomaceae chloroplast genomes, with *A. thaliana* as an outgroup reference, highlights structural variations in IR boundaries. Variability in the positioning of IR boundaries can indicate evolutionary divergence, genomic rearrangements, and potential recombination events among species. The total chloroplast genome size among the analyzed species ranged from 154,124 bp (*Cl. lutea*) to 159,393 bp (*Co. paradoxa*), with *C. houtteana* measuring 157,714 bp, placing it near the middle of the observed range. The IR region lengths also exhibited variation, with *C. houtteana* possessing 25,805 bp IRs, which is similar to *T. hassleriana* (25,804 bp) but shorter than *Co. paradoxa* (26,291 bp) and *C. pallida* (26,209 bp). These differences suggest differential expansion or contraction events that have shaped the genome sizes in Cleomaceae (Figure 5).

The JLB (IRb-LSC) boundary, which separates the LSC region from the inverted repeat B (IRb), was positioned within the *rpl*22 gene in *C. houtteana*, which is consistent with *C. chrysantha*, *C. pallida*, and *T. hassleriana*. However, in *Cl. lutea* and *Cl. serrulata*, this boundary shifted slightly upstream, affecting the length of *rpl*22. This minor shift suggests a lineage-specific trend in IR boundary positioning among Cleomaceae. The LSC region of *C. houtteana* measured 87,506 bp, which is similar to *T. hassleriana* (87,509 bp) but slightly larger than *Cl. serrulata* (83,777 bp), reinforcing the role of IR boundary movement in genome size variability.

The JSB (IRb-SSC) boundary, which marks the transition between the IRb and the SSC region, exhibited differences in the positioning of *ycf*1 and *ndh*F. In *C. houtteana*, *ndh*F extended 1090 bp into the SSC, which is comparable to *C. chrysantha* (1100 bp) but significantly shorter than in *Cl. lutea* (1027 bp) and *Co. paradoxa* (7965 bp). The extended overlap of *ndh*F into the SSC in some species suggests either IR contraction or expansion through genomic rearrangement events (Figure 5). The presence of length variation in *ndh*F at this boundary indicates differences in selective constraints possibly linked to altered functionality in photosynthetic or respiratory pathways.

The JSA (IRa-SSC) boundary, marking the transition between the SSC and inverted repeat A (IRa), displayed significant variation in the positioning of *ycf*1. In *C. houtteana*, *ycf*1 extended 5468 bp into the SSC, a value close to *C. chrysantha* (5411 bp). The presence of expanded *ycf*1 regions in some species suggests that structural changes in the IR region have occurred through gene conversion or recombination events. *Ycf*1 is a well-known hotspot for variation in plastid genomes, and its position within the IR further supports its role in genome plasticity.

The JLA (IRa-LSC) boundary, which delineates the end of the IRa region and the beginning of the LSC, showed a highly conserved placement in *C. houtteana* and related species. The LSC region in *C. houtteana* measured 87,506 bp, nearly identical to *T. hassleriana* (87,509 bp), indicating a stable genome structure between these two species (Figure 5). The location of *rpl*22 and *trn*H-GUG at this boundary remained consistent across species, except for minor variations in the flanking intergenic regions. In *A. thaliana*, the IR boundary was positioned slightly differently, suggesting an ancestral state before Cleomaceae-specific contractions and expansions.

### 2.11. Genome Structure and Inversions in C. houtteana and Related Chloroplast Genomes

The genome alignment visualization generated using PyGenome provides a comparative view of plastid genome synteny and structural rearrangements across Cleomaceae species, with *A. thaliana* included as an outgroup reference. The conserved regions are represented by collinear blocks (brown), while structural rearrangements, including inversions and translocations, are highlighted by crossing connections (green). This analysis reveals significant similarities in chloroplast genome organization among Cleomaceae members, while identifying lineage-specific rearrangements.

The overall genomic architecture among Cleomaceae species appears largely collinear, with strong synteny observed between *C. houtteana*, *C. chrysantha*, *C. pallida*, and *T. hassleriana*. The presence of continuous brown regions across these genomes indicates high sequence conservation, suggesting limited structural modifications in these species. *C. houtteana* exhibits a genome structure closely matching *C. chrysantha*, indicating a shared evolutionary lineage with minimal rearrangements. Similarly, *T. hassleriana* maintains significant genomic collinearity with these species, reinforcing its phylogenetic proximity within Cleomaceae.

**Figure 6:**
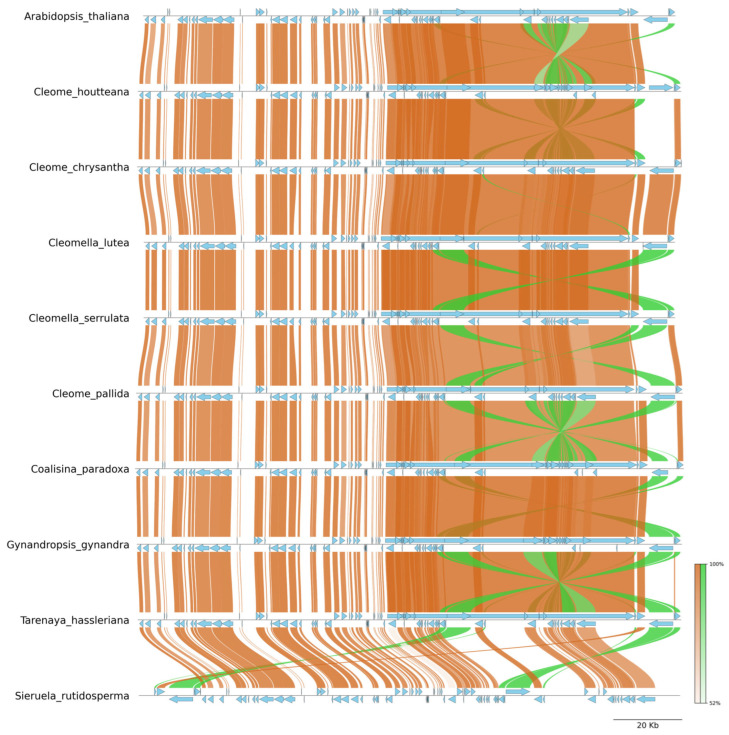
Synteny plot of *C. houtteana* plastome with eleven related species’ plastomes. The synteny plot shows normal links with a chocolate color, inverted links with a lime-green color, and gene features with a sky-blue color.

However, distinct genome inversions are observed in certain species, particularly *Cl. lutea*, *Cl. serrulata*, and *Co. paradoxa* (Figure 6). The large green arcs indicate substantial inverted segments within these genomes, particularly in the IR regions and SSC regions. The presence of large-scale inversions in these species suggests independent evolutionary events that have reshaped genome architecture. These inversions likely resulted from recombination between IR regions, a common phenomenon in plastid genomes that contributes to structural variation.

Compared to *A. thaliana*, all Cleomaceae genomes exhibit structural rearrangements, indicating divergence from the ancestral plastid genome structure. The alignment shows that while core chloroplast genes are conserved, genome evolution in Cleomaceae is accompanied by moderate rearrangements. The most prominent divergence is seen in *S. rutidosperma*, where multiple inversions and translocations are evident in the LSC and SSC regions, suggesting a more complex evolutionary history (Figure 6).

The presence of large inversions in *Cl. lutea*, *Cl. serrulata*, and *Co. paradoxa* suggests lineage-specific genome rearrangements, potentially driven by selective pressures or adaptations to ecological niches. These inversions may influence gene order, expression patterns, and recombination rates, thereby contributing to species differentiation. In contrast, the high synteny among *C. houtteana*, *C. chrysantha*, and *T. hassleriana* suggests strong genome conservation and stability.

### 2.12. Comparative Sequence Divergence Analysis of Cleomaceae Chloroplast Genomes

Chloroplast genome conservation and sequence divergence provide valuable insights into evolutionary relationships, functional adaptations, and structural variations among species. The mVISTA alignment of *C. houtteana* and related Cleomaceae species, with *C. houtteana* serving as the reference genome, reveals a highly conserved plastid genome organization. Despite strong synteny, distinct sequence variations are observed, particularly in intergenic regions, untranslated regions (UTRs), and certain protein-coding genes, highlighting lineage-specific genomic modifications.

**Figure 7:**
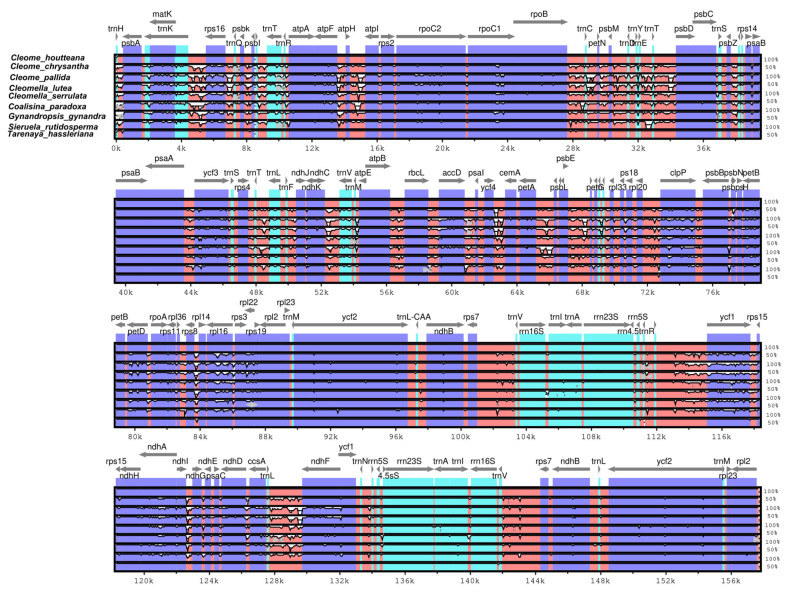
Visual alignment of *C. houtteana* and eight related plastomes from the Cleomaceae family. VISTA-based identity plot showing sequence identity among these species, using *C. houtteana* as a reference. The vertical scale indicates percent identity, ranging from 50 to 100%. The horizontal axis indicates the coordinates within the chloroplast genome. Arrows indicate the annotated genes and their transcription direction.

The analysis indicates that coding regions associated with essential chloroplast functions are highly conserved across all species. Genes involved in photosynthesis (*atp*A, *atp*B, *rbc*L *psa*A, *psa*B, *psb*C, and *psb*D), self-replication (*rps*2, *rps*4, *rps*16, *rpl*2, and *rpl*20), and transcriptional machinery (*rpo*A, *rpo*B, *rpo*C1, and *rpo*C2) exhibit minimal sequence divergence, suggesting strong purifying selection maintaining their functional integrity (Figure 7). Ribosomal proteins and RNA polymerase subunits remain nearly identical in all species, reflecting their indispensable role in plastid gene expression and protein biosynthesis.

In contrast, non-coding regions, including intergenic spacers, introns, and UTRs, show significant sequence divergence, suggesting evolutionary hotspots susceptible to mutational events and structural rearrangements. The most notable divergence is observed in the *trn*H-*psb*A, *trn*K, *mat*K, *mat*K-*rps*16, *ycf*3, *atp*H-*atp*I, *clp*P, *pet*A-*psb*I, *trn*L-*ndh*F, *ycf*1, *rps*16, *rrn*5s-*ycf*1, and *acc*D regions frequently implicated in plastid genome expansion and recombination events (Figure 7). High variability in these regions may contribute to species differentiation and genome plasticity.

One of the most highly divergent genes is *ycf*1, which has been widely recognized as a hotspot for evolutionary change in plastid genomes. *Ycf*1 exhibits considerable sequence variation, particularly in *Co. paradoxa* and *S. rutidosperma*, while *C. chrysantha*, *C. pallida*, and *T. hassleriana* show greater sequence conservation with *C. houtteana*. This pattern is consistent with previous studies identifying *ycf*1 as one of the most variable genes in chloroplast genomes, making it a promising molecular marker for phylogenetic and species differentiation studies. The presence of distinct sequence variations in intergenic regions and specific protein-coding genes underscores the dynamic nature of chloroplast genomes within Cleomaceae. These variations may reflect adaptive responses to environmental pressures or lineage-specific evolutionary trajectories.

### 2.13. Phylogenetic Analysis

**Figure 8:**
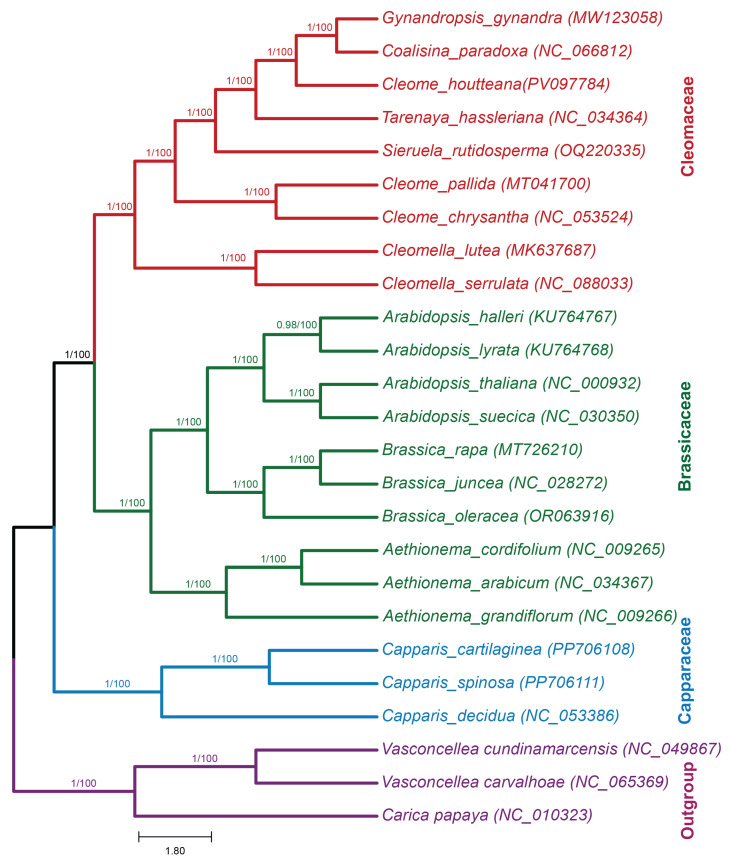
Phylogenetic trees were constructed from the whole-plastome dataset among 25 members of the order Brassicales, representing 12 different genera, using different methods such as Bayesian inference (BI) and Maximum Likelihood (ML). Numbers above the branches are the posterior probabilities of BI and bootstrap values of ML.

A phylogenetic analysis was performed to determine the evolutionary position of *C. houtteana* within the Cleomaceae family. The analysis included eight related species from Cleomaceae, ten species from Brassicaceae, three species from Capparaceae, and three species from Caricaceae as outgroups using whole-chloroplast genomes downloaded from the NCBI database. Two methods were employed: Maximum Likelihood (ML) and Bayesian Inference (BI), with bootstrap values supporting the nodes. The phylogenetic trees generated by ML and BI methods displayed congruent topologies, confirming the monophyletic nature of Cleomaceae. *C. houtteana* grouped closely with *T. hassleriana* and *S. rutidosperma*, with robust bootstrap support (100/100), indicating their recent common ancestry. Within Cleomaceae, *C. houtteana* formed a distinct clade with *C. pallida* and *C. chrysantha*, suggesting a divergence pattern consistent with previous molecular studies (Figure 8). The Brassicaceae species clustered into a well-supported clade with *A. thaliana*, *Brassica rapa*, and *B. oleracea* forming subgroups, all supported by 100% bootstrap values. Capparaceae species (*Capparis spinosa*, *Capparis decidua*, and *Capparis cartilaginea*) were clearly separated, forming an outgroup to Cleomaceae and Brassicaceae. Overall, the results strongly support the placement of *C. houtteana* within the Cleomaceae family, highlighting its evolutionary relationship with closely related species and its divergence from Brassicaceae and Capparaceae lineages. The high bootstrap values from both ML and BI methods further validate the robustness and reliability of these phylogenetic relationships.

## 3. Discussion

The comparative genomic examination involving *C. houtteana* and other Cleomaceae and Brassicales relatives shows both evolutionary conservation and taxon-specific divergence in plastid genomes displayed in angiosperms. The chloroplast genome of *C. houtteana* (157,714 bp) remains within the expected size range of Cleomaceae species. However, the stability of the family is tempered by the increasing variation in genome size, especially in the LSC and SSC regions that are easily affected by evolutionary processes such as expansion and contraction of the IR boundary, which is a common phenomenon in angiosperm plastid genomes [11,16]. These processes soften the structural rigidity of the genomes, fostering both genome plasticity and adaptive evolution in diverse lineages. The GC content (35.8%) is slightly lower than some members of the Brassicaceae family, such as *A. thaliana* (36.3%) and *Brassica napus* (36.2%), but allows for differences in non-coding regions, which is consistent with other Cleomaceae species [17,18].

The chloroplast genome of *C. houtteana* consists of 129 genes, which include 84 coding sequences for proteins, 8 ribosomal RNA genes, and 37 transfer RNA genes. While this is similar to other species in the Cleomaceae family, it is slightly less than *C. pallida*, which has 134 genes. The high conservation across Cleomaceae and Brassicales of critical photosynthetic genes like *rbc*L, *psa*A, *atp*A, *psb*A-*psb*Z, and *ndh*A-*ndh*K indicates significant purifying selection in fundamental functions of the plastid. It is important to mention that some genes containing introns, including *rpl*2 and *rps*16, are crucial for the post-transcriptional regulation of the chloroplasts. Their loss in part in *C. chrysantha* and *Cl. lutea* suggests independent intron losses, which have been noted in some species of the Brassicaceae family like *A. thaliana*.

With regard to the number of genes containing introns, *C. houtteana’s* total of 16 is comparable to *Co. paradoxa* but is more than *C. chrysantha*’s total of 11. This indicates that *C. houtteana* has a more prominent role of introns in the regulation of RNA processing and plastid gene introns. Earlier work from within Cleomaceae has shown that singular ribosomal and NADH dehydrogenase genes containing introns allow the involvement of alternative splicing and the regulation of expression plasticity of the genome [15,19]. This alteration in retention of introns for the Cleomaceae family suggests that some members have experienced particular evolutionary forces pertaining to genome structure and transcriptional activity.

The study of codon usage revealed a strong bias towards A/T-ending codons that is typical among the members of the Cleomaceae family [20]. The most frequent codons (TTT, ATT, and GAA) are also linked to major chloroplast activities, as in the case of Brassicaceae, where post-translational modification is crucial for adequate protein expression [15]. Selective synonymous codon bias indicates optimization of translational efficacy, a characteristic observed in other plastid genomes of Cleomaceae and Brassicales [15,21]. In *C. houtteana*, as in other plastid genomes, there were more palindromic and tandem repeats than non-repeats. The overall number of repeats, as well as the dominance of short repeats, 15–30 bp in length, indicates that these sequences play an important role in the stabilization and recombination of the genome [17,22]. The Brassicaceae species, such as *B. rapa*, which are known to contain large amounts of repeats greater than 50 bp, suggest that members of the Cleomaceae family have a reduced capacity for plastome rearrangement [15,23].

Expansion and contraction events at IR boundaries significantly influence chloroplast genome evolution in angiosperms. In *C. houtteana*, IR boundaries were conserved relative to closely related Cleomaceae species, suggesting genome stability [23,24]. However, minor shifts in IR boundaries in *Cl. serrulata* and *Cl. lutea* resemble patterns observed in other Brassicales members, such as *Cardamine* species, where recombination-induced IR boundary movements drive genome size variability [25,26]. The extension of *ycf*1 into IR regions suggests a functional role in genome stability. *ycf*1 is known as a hypervariable gene in angiosperm plastid genomes and is often used as a phylogenetic marker [26,27]. Its observed variability in *Co. paradoxa* and *S. rutidosperma* further supports its role in plastid genome evolution.

The Ka/Ks analysis revealed that most *C. houtteana* chloroplast genes are under strong purifying selection, which is consistent with other angiosperms [28,29]. However, *acc*D, *cem*A, and *clp*P exhibited higher Ka/Ks ratios, suggesting relaxed selection and potential functional divergence. The *acc*D gene, which plays a key role in chloroplast lipid biosynthesis, has shown signs of adaptive evolution in Brassica species, where it has undergone modifications linked to lipid metabolism and plastid stability [30,31]. Similarly, *clp*P, essential for protein degradation and stress response, displayed high Ka/Ks values across Cleomaceae, with *Cl. lutea* reaching 1.210. However, Fisher’s Exact Test did not yield statistical significance, indicating that while relaxed selection is likely, strong positive selection remains inconclusive [28,29]. These findings suggest that while *clp*P and *acc*D may undergo functional shifts, further experimental validation is required to confirm their potential adaptive roles. Future studies involving gene expression analysis, biochemical assays, or mutational studies could clarify whether these genes contribute to chloroplast adaptation and genome stability in *C. houtteana* and its relatives.

The nucleotide diversity (Pi) analysis of the *C. houtteana* chloroplast genome revealed distinct variation patterns. High diversity was observed in non-coding regions, particularly in intergenic spacers (*trn*H–*psb*A and *ndh*F–*rpl*32) and hypervariable loci (*ycf*1 and *ycf*2), marking them as evolutionary hotspots. Conversely, coding regions, such as *rbc*L, *psa*A, *psb*B–*psb*D, and *ndh*C, showed low diversity, indicating strong purifying selection. These patterns align with previous studies on Cleomaceae and Brassicaceae, highlighting functional region conservation and non-coding region variability [11,26]. The pronounced variability in *ycf*1 further supports its potential as a molecular marker for species identification and phylogenetics. Overall, these findings underscore the evolutionary dynamics shaping chloroplast genomes within Cleomaceae.

The phylogenetic analysis of *C. houtteana* using Maximum Likelihood (ML) and Bayesian Inference (BI) methods based on whole-chloroplast genomes provided valuable insights into its evolutionary placement within Cleomaceae. Our results align with those of Patchell et al. (2014) [1], confirming that *C. houtteana* clusters closely with *T. hassleriana* and *G. gynandra*, supporting its reclassification under the genus Tarenaya. This relationship highlights a shared evolutionary lineage, reinforcing previously proposed taxonomic revisions. Additionally, our findings concur with Tamboli et al. (2016) [32], demonstrating a clear separation of Corynandra and Cleoserrata from the Cleome clade, suggesting distinct evolutionary lineages within Cleomaceae. Our results also align with the phylogenomic study by Feodorova et al. (2010) [4], which used combined chloroplast, mitochondrial, and nuclear ribosomal DNA to reconstruct the evolutionary history of Cleomaceae, placing *C. houtteana* in close proximity to Tarenaya. Compared to Vasquez et al. (2024) [33], who employed ITS markers, our whole-chloroplast genome approach provided stronger node support with higher Bayesian posterior probabilities. Hall et al. (2002) [2] further supported our results by showing clear divergence between Cleomaceae and Brassicaceae through chloroplast sequence analysis. Overall, our findings provide a robust phylogenetic framework for Cleomaceae, emphasizing congruence and advancements compared to previous studies. The integration of whole-plastome data enhances the resolution of species relationships, contributing to a deeper understanding of Cleomaceae evolution.

## 4. Materials and Methods

Fresh leaves were collected from *C. houtteana* plants cultivated at the Agriculture Research Center, under the supervision of the District Director of Agriculture Extension, Mardan, Khyber Pakhtunkhwa, Pakistan (34.18202° N, 72.04449° E). A total of five specimens were sampled to ensure representative biological replication. The samples were immediately placed in liquid nitrogen and subsequently stored at −80 °C. A voucher specimen (CHN-CH5) was deposited at the Herbarium Center of the Agriculture Research Center, KPK, Pakistan. The species identification was confirmed by Dr. Muhammad Waqas, an Agronomist at the Agriculture Research Center, KPK, Pakistan. Sample collection and processing were conducted in compliance with national policies and regulations, and permission was granted by the Environmental Protection Agency, Khyber Pakhtunkhwa, Pakistan (Permit No. CH653/13/18).

### 4.1. DNA Extraction and Sequencing

In order to obtain optimal DNA samples from the collected leaf samples of *C. houtteana*, a detailed and stepwise approach was employed. First, the leaves were thoroughly pulverized into a powder form in the presence of liquid nitrogen, which aids in the effective release of the DNA from the cells. For the isolation of DNA, we employed the DNeasy Plant Mini Kit from Qiagen in Valencia, CA, USA, which is known for its efficiency. The kit effortlessly extracted the DNA from the plant samples and, accompanied by the protocol, ensured high-grade quality DNA. After the isolation of DNA was carried out, the next step we performed was sequencing chloroplast DNA, which was accomplished using the Illumina HiSeq-2000 platform at Macrogen located in Seoul, Republic of Korea. This equipped sequencing platform was capable of generating high quantities of raw reads for *C. houtteana*, specifically in the order of 878,620,821 raw reads. However, the sequences still required refinement so as to ensure they were reliable and accurate, which is the reason for the implemented filtering. To do this, we set a rigid filtering criterion based on a Phred score of less than thirty, which enabled us to purge all reads not attaining the desired threshold. This quality control step ensured the retention of high-quality sequences for further examination.

We assembled the plastome using GetOrganelle (version 1.7.5) [34] and SPAdes (version 3.10.1) (http://bioinf.spbau.ru/spades, accessed on 10 November 2024) to ensure accuracy and consistency. GetOrganelle, designed specifically for organelle genome assembly, was run with k-mer sizes of 21, 45, 65, 85, and 105 to optimize contig formation. A minimum read depth of 20× was applied to filter low-confidence sequences. For validation, we also used SPAdes with the metaSPAdes module, incorporating k-mer sizes of 21, 33, 55, 77, and 99 to improve assembly quality. We set a minimum coverage threshold of 30× to remove potential chimeric sequences. Finally, both assemblies were compared using BLAST (version 2.16.0) searches against reference plastomes, and raw reads were mapped back to confirm accuracy and completeness.

### 4.2. Genome Annotation

Plastome annotation was achieved in multiple steps using various tools and software. The first annotations were performed using the online genome annotation tools CpGAVAS2 (version 2.0) [35] and GeSeq (version 2.03) (https://chlorobox.mpimp-golm.mpg.de/geseq.html, accessed on 10 November 2024). In addition, tRNA genes in the plastomes were annotated using the well-known program tRNAscan-SE (version 1.21) [36]. The accuracy of the annotations was verified through a comparative analysis of plastomes against reference genomes using Geneious Pro (version 10.2.3) [37] and tRNAscan-SE (version 1.21) [36]. Manual curation was performed to resolve ambiguities in gene regions by cross-referencing with well-annotated plastomes, ensuring accurate start and stop codons, and refining intron–exon boundaries. Potential frame shifts, pseudogenes, and misidentified tRNA genes were corrected by analyzing sequence alignments, conserved structural features, and homologous gene patterns. Adjustments were made where necessary to improve annotation accuracy, and all curated annotations were validated against publicly available plastome databases to ensure consistency and reliability. Chloroplot was employed for the visualization of the plastome’s morphological features [38]. In addition, genetic divergence was analyzed with mVISTA using the shuffle-LAGAN mode, with the plastome of *C. houtteana* as a reference [39]. The mean pairwise sequence divergence of the *C. houtteana* plastome with ten other species (*C. chrysantha*, *C. pallida*, *Cl. lutea*, *Cl. serrulata*, *Co. paradoxa*, *G. gynandra*, *S. rutidosperma*, and *T. hassleriana*) was established.

Analysis of gene order and multiple sequence alignment was carried out for comparative sequence analysis in order to identify leftover or ambiguous gene annotations. In order to carry out whole-genome alignment, MAFFT (version 7.222) with the default settings was employed [40]. The pairwise sequence divergence was computed based on the Kimura two-parameter (K2P) model. This method was effective for evaluating genetic information. The analysis was conducted with the aid of the DnaSP software (version 6.13.03) [41] which carried out the sliding window analysis with a window of 600 bp and a step of 100 bp. Through this analysis, we determined the variation in nucleotides, particularly the nucleotide diversity (Pi). The Heatmap2 package within R-software (version 4.4.3) was employed to display the divergence of genes and shared genes among plastomes of different species. Furthermore, with the pyGenomeViz package (version 0.2.1), we made a synteny plot by employing the pgv-mmseqs mode and an identity threshold of 50%. The corresponding reference for pyGenomeViz is available on GitHub at the following link: https://github.com/moshi4/pyGenomeViz, accessed on 10 November 2024.

We analyzed Ka/Ks ratios for shared chloroplast genes in Cleomaceae using *C. houtteana* as a reference, which were calculated via the KaKs Calculator in TBtools (version 1.112) [42]. Ka/Ks < 1 indicates purifying selection, =1 suggests neutral evolution, and >1 implies potential positive selection. To ensure statistical validity, we applied Fisher’s Exact Test to genes with Ka/Ks > 1, using non-synonymous (cN) and synonymous (cS) substitution counts. Only genes with *p* < 0.05 were considered to be under strong positive selection. Genes with Ka/Ks > 1 but non-significant *p*-values were interpreted as relaxed selection or functional divergence rather than confirmed adaptive evolution. All analyses were performed in Python (version 3.12.2) (SciPy package), and the results were incorporated into our discussion.

### 4.3. Characterization of Repetitive Sequences and SSRs

In the plastome of *C. houtteana* and eight other closely related members of the Cleomaceae family, a series of repetitive sequences were identified and categorized into three types: forward (direct) repeats, reverse repeats, and palindromic repeats. These classifications were based on the definitions provided by REPuter [43], a web-based tool used for repeat analysis. To ensure the accurate detection of repetitive sequences, we set the minimum repeat size to 8 base pairs and limited the maximum number of computed repeats to 50. Additionally, we used a Hamming distance of 0, meaning only exact matches were considered, and applied a sequence alignment method that excluded mismatches to enhance specificity. These parameters were carefully selected to capture both short and long repeats while maintaining high accuracy and biological relevance in the analysis. In the same way, the software MISA (version 2.2) [44] was employed for measuring SSRs. The parameters employed for this purpose are as follows: for one base pair repeat, ≥8 repeat units; for two base pair repeats, ≥6 repeat units; for three and four base pair repeats, ≥4 repeat units; and for five and six base pair repeats, ≥3 repeat units. In addition, the online tool Tandem Repeats Finder (version 4.09) was employed for tandem repeat calculations [45].

### 4.4. Genome Divergence

*C. houtteana* and closely related species were examined for possible differences in the complete plastome as well as the shared protein-coding genes. Multiple sequence alignment was employed for comprehensive comparative analysis in order to enhance ambiguous and deficient gene annotation quality. A comparative analysis was conducted with the aid of multiple sequence alignment, in which the analysis and examination of gene order were performed to improve the quality of ambiguous and deficient gene annotations. Plastome annotations were performed by employing MAFFT (version 7.222) with the default settings [40] using default values. Estimates for pairwise sequence divergence were computed by employing Kimura’s two-parameter model (K2P) methodology [40]. We generated a synteny plot with the pgv-mmseqs mode. For this purpose, the identity threshold was adjusted to 50% with the help of pyGenomeViz (version 0.2.1), the relevant source for which is available on Github at the following URL: https://github.com/moshi4/pyGenomeViz, accessed on 10 November 2024.

### 4.5. Phylogenetic Analyses

For insights into the phylogenetic position of *C. houtteana* within Cleomaceae, eight published plastome sequences of Cleomaceae, ten species from Brassicaceae, three species from Capparaceae, and three species from Caricaceae (outgroup) were downloaded from the NCBI database. A detailed analysis was carried out by utilizing a whole-genome dataset. The downloaded nucleotide sequences were aligned and combined with the help of MAFFT, keeping the settings at default as per reported protocols [40]. jModelTest 2, as reported by [46], was employed to determine the nucleotide evolution’s best fitting model, i.e., TVM + F + I + G4. For the deduction of the phylogenetic relationship among *C. houtteana* and related species, two different approaches, i.e., Bayesian Inference (BI) and Maximum Likelihood (ML) trees, were used. The BI tree was built with MrBayes (version 3.12) software using the MCMC sampling algorithm. Second, an ML tree was created using PAUP* 4.0. The ML tree was constructed with 1000 bootstraps that provided support values at different nodes. For the BI analysis, four chains were used: three were heated chains and one was a cold chain. These were run for 10 million generations, sampling every thousand prints and printing every 10 thousand samples. To make sure there was convergence, a burn-in of 2500, which is 25 percent of the total number of generations divided by the sampling frequency, was used. Finally, a 50% majority-rule consensus tree was derived from the phylogenetic trees generated, and Figtree [47] was employed for the visual representation of the relationship between *C. houtteana* and related species. The visual representation was based on the whole plastomes of *C. houtteana* and related species.

## 5. Conclusions and Future Directions

This study reports the first complete chloroplast genome of *C. houtteana*, providing valuable insights into its genomic architecture and evolutionary relationships within Cleomaceae. The 157,714 bp genome, with 129 annotated genes, reveals key features such as conserved photosynthesis genes, SSRs, and divergence hotspots (*ycf1* and *ycf2*), which can serve as molecular markers for phylogenetic analysis. Comparative genomics highlights variations in IR boundaries and adaptive evolution in genes (*acc*D, *cem*A, and *clp*P), supporting species-specific functional divergence. Phylogenetic analysis confirms *C. houtteana*’s close relationship with *T. hassleriana* and *S. rutidosperma*, clarifying its taxonomic placement within Cleomaceae.

Future research should focus on population genetics using identified SSR markers to assess genetic diversity and support conservation strategies. Functional studies of adaptive genes through transcriptomics and genome editing (e.g., CRISPR/Cas9) will enhance the understanding of stress responses. However, given the current limitations in chloroplast genome engineering in *C. houtteana*, genome editing applications may be more feasible for nuclear or mitochondrial genes rather than the chloroplast genome. Further research on optimizing chloroplast transformation techniques in Cleomaceae could overcome these challenges in the future. Additionally, expanding phylogenetic analysis with more Cleomaceae species and using *ycf*1 and *ycf*2 for species barcoding will refine taxonomic classifications. Further investigations into chloroplast genome evolution, including IR boundary shifts, codon usage, and RNA editing patterns, will advance our understanding of Cleomaceae evolution. This study lays a foundation for future research, supporting taxonomy, conservation, and biotechnological applications.

## Figures and Tables

**Table 1:** Summary of all *C. houtteana* and related plastomes.

	Genome Size	% GC	LSC Size	SSC Size	IR Size	Number of Total Genes	Protein-Coding Genes	rRNA Genes	PCD Size	Genes with Introns
*C. houtteana*	157,714	35.8	87,506	18,598	25,805	129	84	8	76,590	16
*C. chrysantha*	158,111	36	87,162	18,425	26,251	133	86	8	79,488	11
*C. pallida*	158,576	35.8	87,683	18,420	26,264	134	87	8	80,076	13
*Cl. lutea*	154,124	36.5	83,700	18,114	26,155	132	87	8	78,444	15
*Cl. serrulata*	154,226	36.5	83,777	18,119	26,156	131	85	8	78,567	15
*Co. paradoxa*	159,393	35.8	88,191	18,620	26,291	130	85	8	73,353	16
*G. gynandra*	158,152	35.8	87,019	18,548	26,181	129	85	8	79,134	15
*S. rutidosperma*	157,073	36.0	86,423	18,485	26,083	132	86	8	79,731	14
*T. hassleriana*	157,688	35.8	87,509	18,571	25,804	131	85	8	79,755	14

C. = Cleome; Cl. = Cleomella; Co. = Coalisina; G. = Gynandropsis; S. = Sieruela; T. = Tarenaya.

**Table 2:** Genes in the sequenced *C. houtteana* plastome.

Category of Genes	Group of Genes	Name of Genes
Genes for photosynthesis	Subunits of ATP synthase	*atp*A, *atp*B, *atp*E, *atp*F, *atp*H, *atp*I
Genes for photosynthesis	Subunits of photosystem II	*psb*A, *psb*B, *psb*C, *psb*D, *psb*E, *psb*F, *psb*I, *psb*J, *psb*K, *psb*L, *psb*M, *psb*N, *psb*T, *psb*Z, *ycf*3
Genes for photosynthesis	Subunits of NADH dehydrogenase	*ndh*A, *ndh*B, *ndh*B, *ndh*C, *ndh*D, *ndh*E, *ndh*F, *ndh*G, *ndh*H, *ndh*I, *ndh*J, *ndh*K
Genes for photosynthesis	Subunits of cytochrome b/f complex	*pet*A, *pet*B, *pet*D, *pet*G, *pet*L, *pet*N
Genes for photosynthesis	Subunits of photosystem I	*psa*A, *psa*B, *psa*C, *psa*I, *psa*J
Genes for photosynthesis	Subunit of Rubisco	*rbc*L
Self-replication	Large subunit of ribosome	*rpl*14, *rpl*16, *rpl*2, *rpl*2, *rpl*20, *rpl*22, *rpl*23, *rpl*23, *rpl*33, *rpl*36
Self-replication	DNA-dependent RNA polymerase	*rpo*A, *rpo*B, *rpo*C1, *rpo*C2
Self-replication	Small subunit of ribosome	*rps*11, *rps*12, *rps*12, *rps*14, *rps*15, *rps*16, *rps*18, *rps*19, *rps*2, *rps*3, *rps*4, *rps*7, *rps*7, *rps*8
Other genes	Subunit of Acetyl-CoA-carboxylase	*acc*D
Other genes	C-type cytochrome synthesis gene	*ccs*A
Other genes	Envelop membrane protein	*cem*A
Other genes	Protease	*clp*P
Other genes	Maturase	*mat*K
Unknown	Conserved open reading frames	*ycf*1, *ycf*2, *ycf*3, *ycf*4

**Table 3:** The genes with introns in the *C. houtteana* plastome and the length of exons and introns.

Gene	Strand	Start	End	ExonI	IntronI	ExonII	IntronII	ExonIII
*trn*K-UUU	−	1777	4412	37	2564	35		
*rps*16	−	5521	6665	40	878	227		
*trn*T-CGU	+	9285	10,084	34	723	43		
*atp*F	−	12,218	13,502	145	730	410		
*rpo*C1	−	21,561	24,368	432	765	1611		
*ycf*3	−	44,239	46,256	124	707	230	804	153
*trn*L-UAA	+	48,857	49,480	35	539	50		
*trn*V-UAC	−	53,137	53,834	39	624	35		
*clp*P	−	72,810	74,906	71	901	294	596	235
*rpl*16	−	84,438	85,958	9	1113	399		
*ycf*1	+	115,123	117,747	2224	57	344		
*ndh*A	+	119,738	121,946	553	1126	530		
*trn*A-UGC	−	137,847	138,720	37	801	36		
*trn*E-UUC	−	138,785	139,804	32	948	40		
*ndh*B	+	145,080	147,300	775	682	764		
*rpl*2	+	156,039	157,549	391	686	434		
*pet*B	+	77,895	79,347	6	805	642		
*pet*D	+	79,545	80,770	8	743	475		

## Data Availability

The data presented in the study were deposited in the National Center for Biotechnology Information (NCBI) repository, accession number PV097784.

## Supplementary Materials

The following supporting information can be downloaded at https://www.mdpi.com/article/10.3390/ijms26083527/s1.
